# Editorial: Examining genetic and epigenetic regulation in cardiovascular development, regeneration and disease

**DOI:** 10.3389/fcvm.2023.1306263

**Published:** 2023-10-18

**Authors:** Eltyeb Abdelwahid, Katherine Athayde Teixeira de Carvalho

**Affiliations:** ^1^ Feinberg Cardiovascular Research Institute, Feinberg School of Medicine, Northwestern University, Chicago, IL, United States; ^2^ Department of Medicine, University of Illinois at Chicago, Chicago, IL, United States; ^3^ Advanced Therapy and Cellular Biotechnology in Regenerative Medicine Department, The Pelé Pequeno Príncipe Research Institute, Child and Adolescent Health Research and Pequeno Príncipe Faculties, Curitiba, Brazil

**Keywords:** genetic, epigenetic, cardiovascular, disease, therapy

**Editorial on the Research Topic** Examining genetic and epigenetic regulation in cardiovascular development, regeneration and disease

Heart disease is the leading cause of death worldwide and is associated with significant socio-economic problems. Examining genetic and epigenetic regulation in cardiovascular development, regeneration and disease can explain multifaceted mechanisms responsible for cardiac dysfunction. Targeting these mechanisms is hoped to ameliorate heart diseases by offering appropriate therapeutic solutions. This special issue comprises 9 articles, including 5 original papers and 4 reviews, which cover pivotal areas including atherosclerosis, atrial fibrillation, fibrosis, cardiac remodeling, therapeutic vaccination, non-coding RNA, thrombosis, and hypertrophic cardiomyopathy. Thus, multiple questions are addressed to draw attention to the current knowledge in this field and to open the door for possible future research advancing molecular genetics and epigenetics of cardiac pathologies and related interventions.

As reviewed by Zhao et al. FGF21, a promising cardioprotective factor, has been shown by recent studies to improve cardiac function by playing regulatory roles targeting different heart tissue components, including cardiac myocytes, immune cells, and fibroblasts. These effects occur via modulation of distinct biological mechanisms, including cell death, metabolism, oxidative stress, fibrosis, and inflammatory processes. Thus, FGF21 appears to be a potential protective molecule in the heart. Xue et al. updated the readers on works addressing the roles of non-coding RNA in atrial fibrillation. They discussed the concept of ncRNAs as strong regulators of the occurrence and progression of AF. Iversen et al. described AtheroVax as a peptide vaccine targeting sTNFR2 to inhibit the progression of atherosclerosis. The authors expect this novel vaccine to have a longer duration of action compared to current therapies which require chronic treatment that encounters problems associated with lack of patient compliance. Matta et al. explored potential differences in cardiovascular outcomes in heterozygous familial hypercholesterolemia (HFH) population under medical care with vs. without a causative variant and assessed the association between different gene variants and atherosclerotic cardiovascular disease (ASCVD). It appears that the presence of a causative variant may not represent an independent predictor of adverse cardiovascular outcomes in heterozygous familial hypercholesterolemia (HFH) patients. The elevation of LDL-c level is suggested to remain the strongest independent predictor of ASCVD. Doh et al. discussed various existing and emerging strategies to improve technologies concerning variants of uncertain significance (VUS) and their possible involvement in the pathogenicity of VUS in hypertrophic cardiomyopathy (HCM). The classification of a genetic variant leads to ambiguity in explanation, risk stratification, and clinical setting. Therefore, this review offered information on some basic science methods to help the characterization of VUS in HCM. Kukida et al. manipulated components of the renin angiotensin system in renal proximal tubules to understand if this can alter atherosclerosis in hypercholesterolemic mice. This study found that while whole-body AT1R inhibition reduced atherosclerosis equivalently in the studied male and female mice; PTC-specific manipulation of the RAS pathway did not change hypercholesterolemia-induced atherosclerosis. Wu et al. performed LC–MS/MS analysis to identify proteins with altered ubiquitination in AF tissues. The authors identified significant alterations in ubiquitination between the SR and AF groups. The results characterized alterations that are suggested to modulate the development of AF and may thus provide an effective therapeutic strategy against AF. The study by Nguyen et al. showed a novel mechanism regulating the suppressive effects of L-flow on endothelial cell inflammation, migration, proliferation, apoptosis, and fibrosis via increasing CHK1-induced SENP2 S344 phosphorylation. The findings are considered a good basis for further studies aiming to characterize the potential impact of this mechanism on the cardiovascular system. The study by Natae et al. revealed that the five strongly associated SNPs combined with non-genetic factors may allow the prediction of individual Venous thrombosis (VT) risk susceptibility. These novel findings shed new light on the determinants of VT as one of the three leading problems associated with cardiovascular pathology.

Elucidation of the genetic and epigenetic basis of cardiac disease should pave the way for the identification of new targets and the development of new drugs (1–3). Papers included in this special issue address recent discoveries of mechanisms controlling cardiovascular diseases and discuss their potential to be used in preventive, diagnostic and therapeutic technologies for managing heart dysfunction. Future studies would benefit from using various genetic models and cutting edge molecular and cellular technologies to advance current discoveries by clarifying genetic hubs governing cardiac pathophysiology. We are delighted to have been able to put together in this issue some of the important works reflecting the advances in this field, with much more to come.
